# Galectin-3 inhibition using novel inhibitor GR-MD-02 improves survival and immune function while reducing tumor vasculature

**DOI:** 10.1186/2051-1426-3-S2-P306

**Published:** 2015-11-04

**Authors:** Stefanie Linch, Melissa J Kasiewicz, Michael McNamara, Ian Hilgart, Mohammad Farhad, William Redmond

**Affiliations:** 1 Earle A. Chiles Research Institute/Providence Health and Services, Portland, OR, USA; 2 Earle A. Chiles Research Institute, Providence Cancer Center, Portland, OR, USA

## 

Immunosuppression and reduced cytolytic function of tumor-infiltrating lymphocytes are major obstacles to creating effective therapies for patients. It is known that Galectin-3 (Gal3), a lectin family member, is expressed and secreted by numerous cancers and immune cell subsets. Serum Gal3 expression is higher in patients with metastatic versus non-metastatic disease, and is associated with reduced survival in metastatic melanoma. Furthermore, Gal3 has been implicated in disease progression via the promotion of angiogenesis and metastasis. Interestingly, extracellular Gal3 inhibits the function of tumor-infiltrating lymphocytes (TIL) by associating with and restricting movement of the TCR. Gal3 has also been shown to promote M2 polarization in macrophages and mobilize myeloid cells from the bone marrow to promote a metastatic niche within the tumor. We hypothesized that Gal3 inhibition would improve TIL function while inhibiting the growth and metastasis of tumors. Through collaboration with Galectin Therapeutics, we tested Gal3 inhibition in cancer using the novel Gal3 inhibitor, GR-MD-02, with agonist anti-OX40 therapy. We observed that Gal3 inhibition with GR-MD-02 promoted antigen specific T cell expansion in vivo. Moreover, Gal3 inhibition/OX40 agonism improved survival in the MCA-205 sarcoma and 4T-1 mammary carcinoma models, and prolonged survival in the TRAMP-C1 prostate cancer model. Importantly, Gal3 inhibition/OX40 agonism reduced lung metastases in the 4T-1 model. Within the tumor, we observed an increase in the number of proliferating and IFNg-producing TIL. Gal3 inhibition/OX40 agonism was also associated with a decrease in functional tumor vasculature, as determined by CD31 staining by IHC within the tumors. These studies, along with clinical data from a liver fibrosis trial, supported testing GR-MD-02 in combination with ipilimumab in a Phase I trial for patients with metastatic melanoma.

